# Host–Pathogen Interactions of *Chlamydia trachomatis* in Porcine Oviduct Epithelial Cells

**DOI:** 10.3390/pathogens10101270

**Published:** 2021-10-01

**Authors:** Amanda F. Amaral, Bryan E. McQueen, Kimberly Bellingham-Johnstun, Taylor B. Poston, Toni Darville, Uma M. Nagarajan, Caroline Laplante, Tobias Käser

**Affiliations:** 1 Department of Population Health and Pathobiology, College of Veterinary Medicine, North Carolina State University, 1060 William Moore Drive, Raleigh, NC 27607, USA; afamaral@ncsu.edu; 2 Comparative Medicine Institute, North Carolina State University, 1060 William Moore Drive, Raleigh, NC 27607, USA; 3 Department of Microbiology and Immunology, University of North Carolina, 116 Manning Drive, Chapel Hill, NC 27599, USA; bryanem@email.unc.edu (B.E.M.); lad@email.unc.edu (T.D.); 4 Department of Molecular Biomedical Sciences, College of Veterinary Medicine, North Carolina State University, 1060 William Moore Drive, Raleigh, NC 27607, USA; ksbellin@ncsu.edu (K.B.-J.); claplan@ncsu.edu (C.L.); 5 Department of Pediatrics, University of North Carolina, 116 Manning Drive, Chapel Hill, NC 27599, USA; tbposton@email.unc.edu (T.B.P.); nagaraja@email.unc.edu (U.M.N.)

**Keywords:** *Chlamydia trachomatis*, porcine oviduct epithelial cell, developmental cycle, innate immune response, claudin-4, tight junctions

## Abstract

*Chlamydia trachomatis* (*Ct*) causes the most prevalent bacterial sexually transmitted disease leading to ectopic pregnancy and infertility. Swine not only have many similarities to humans, but they are also susceptible to *Ct*. Despite these benefits and the ease of access to primary tissue from this food animal, in vitro research in swine has been underutilized. This study will provide basic understanding of the *Ct* host–pathogen interactions in porcine oviduct epithelial cells (pOECs)—the counterparts of human Fallopian tube epithelial cells. Using NanoString technology, flow cytometry, and confocal and transmission-electron microscopy, we studied the *Ct* developmental cycle in pOECs, the cellular immune response, and the expression and location of the tight junction protein claudin-4. We show that *Ct* productively completes its developmental cycle in pOECs and induces an immune response to *Ct* similar to human cells: *Ct* mainly induced the upregulation of interferon regulated genes and T-cell attracting chemokines. Furthermore, *Ct* infection induced an accumulation of claudin-4 in the *Ct* inclusion with a coinciding reduction of membrane-bound claudin-4. Downstream effects of the reduced membrane-bound claudin-4 expression could potentially include a reduction in tight-junction expression, impaired epithelial barrier function as well as increased susceptibility to co-infections. Thereby, this study justifies the investigation of the effect of *Ct* on tight junctions and the mucosal epithelial barrier function. Taken together, this study demonstrates that primary pOECs represent an excellent in vitro model for research into *Ct* pathogenesis, cell biology and immunity.

## 1. Introduction

*Chlamydia trachomatis* (*Ct*) causes the most prevalent bacterial sexually transmitted disease worldwide [1]. In many cases, patients remain asymptomatic and therefore do not receive treatment [2]. Untreated *Ct* infections can reach the upper genital tract and infect epithelial cells in the Fallopian tubes; here, infection can lead to ectopic pregnancy, infertility, and chronic pelvic pain [3]. Research on this infection process and the induced immune response is largely performed in mice and humans and both have strongly contributed to our understanding of the host–pathogen interactions during *Ct* infections. *Chlamydia trachomatis* is an intracellular bacterium with a biphasic developmental cycle characterized by two forms of chlamydia particles—infectious, non-replicative elementary bodies (EBs), and non-infectious, replicative reticulate bodies (RBs) [4]. The *Ct* developmental cycle has been well-described in mice [5,6] and humans cells [7,8,9,10,11]; and it has been nicely summarized over the years [4,12,13,14]. This cycle can be divided into five steps: (i) the EB infects the host cell, mainly epithelial cells, via host receptors such as the protein disulfide isomerase (PDI), or the ephrin receptor A2 (EPHA2) [14]; (ii) within 2 h post infection (hpi), it differentiates in an “initial body” into an RB; (iii) thereafter, the RB divides by binary fission in an “inclusion” [14]; (iv) at ~18 hpi, the RB associates with the inclusion membrane and re-differentiates into EBs; (v) by 40–48 hpi, these EBs are released from the host cells by lysis or extrusion to start another round of infection [4]. The infected epithelial cell reacts by initiating an innate immune response that includes the production of immune effectors like chemokines and cytokines for the recruitment and activation of immune cells against infection [15,16].

While these murine and human studies show the valuable contributions of these models, they also have their limitations. Studies on human tissue are limited by the access to tissue and cells; mice on the other hand are easily accessible but they have three major limitations: (i) based on their small size tissue samples are limited; (ii) infections with relevant *Ct* strains are easily cleared; and (iii) the murine immune mechanisms differ from humans, including the arguably most important cytokine in the anti-*Ct* response—interferon (IFN)-γ [17,18,19,20,21]. In contrast to human cells, IFN-γ does not activate the expression of indoleamine 2,3-dioxygenase (IDO) in murine epithelial cells; instead, mice have redundant mechanisms including nitric oxide synthase and others to deplete the intracellular tryptophan pools [22]. This tryptophan depletion is an important mechanism to limit *Chlamydia* propagation through tryptophan starvation [4,23,24,25]. Swine could be used as an alternative animal model that may overcome the limitations observed in murine and human models: swine have three main advantages as a biomedical animal model—easy access to tissue, biological similarities to humans, and susceptibility to *Ct*. First, swine are used as a food animal model: therefore, porcine blood and tissues like genital tracts are by-products of meat production; thus, primary immune and other tissue cells can be collected at slaughter plants at nearly no cost and without the need to sacrifice animals for research. This is a strong advantage in accordance with the 3R principle—replacement, refinement, and reduction [26]. Second, in many aspects including their hormonal cycle, physiology, and immune system, swine are highly similar to humans [27,28,29]. Thirdly, swine are not only susceptible to *Ct*, but they are also the natural host to *Chlamydia suis* (*Cs*)—a close relative to *Ct* [29,30,31,32,33,34,35,36]. Based on these advantages, swine and minipigs have greatly contributed to the study of human *Ct* infections [33]; however, most studies have been in vivo investigations of *Ct* vaccination [30,31,35,36,37,38,39,40] or the analysis of the adaptive immune response to *Ct* [32]. Yet, swine have rarely been used for in vitro studies on chlamydia pathogenesis and host–pathogen interactions. This lack of in vitro studies in porcine tissue represents a great underutilization of this valuable biomedical animal model.

So far, Käser et al. used a porcine ocular cell line (VIDO-R1) to study host–pathogen interactions during ocular chlamydia infections [41]: They showed that the *Cs* developmental cycle in these VIDO-R1 cells was completed within 48 h; and infection induced a complex, and mainly pro-inflammatory immune response. In addition, Guseva et al. studied the hormonal influence on *Ct* infections in primary cultures of porcine genital tract epithelial cells [42]. They showed that *Ct* can infect epithelial cells isolated from the porcine cervix, uterus and uterine horns; and while the toolbox for molecular research in swine is still limited, they concluded that “primary swine genital epithelia cultured ex vivo appear to be an excellent cell model for dissecting the hormonal modulation of several aspects of chlamydial pathogenesis and infection” [42].

The aim of this study was to assess the suitability of porcine oviduct epithelial cells (pOECs) for in vitro investigations of *Ct* infections. These pOECs are the counterparts to human Fallopian tube epithelial cells. Since scarring of the Fallopian tubes are sequelae of chronic *Ct* infections, these cells are arguably the most relevant genital tract epithelial cells for studying *Ct* pathogenesis. To fully establish pOECs for studying host–pathogen interactions of *Ct*, pOECs were exposed to *Ct* and examined for up to 72 hpi for analysis of both the *Ct* developmental cycle (Aim 1) and the pOEC immune response (Aim 2). During the study of the *Ct* developmental cycle, we also noticed an interesting phenomenon: during the active phase of *Ct* replication, the tight junction protein claudin-4 (CLDN-4) appeared diminished from the pOEC membrane. Claudins including CLDN-4 are heavily regulated and key structural and functional components of tight junctions: thereby, they play an integral part in the regulation of the tight junction barrier function [43,44]. While the full range of interactions during CLDN-4 trafficking and signaling within the tight junctions is not fully understood, it has been shown that CLDN-4 interacts with a plethora of molecules during trafficking and tight junction expression [45,46]. Since tight junctions play a key role in maintaining the integrity of the mucosal epithelium, a disruption of tight junction formation could lead to impaired barrier function. Other pathogenic bacteria and their toxins like the *Clostridium perfringens* enterotoxin have been shown to bind CLDN-4 to reduce the tight junction barrier function [44]. For *Chlamydia*, not only has *Ct* been shown to impact epithelial integrity in mice and humans [47,48] but *Ct* infection has also been linked to an increased susceptibility to co-infections such as HIV [49,50,51,52]. Of note, Zhang et al. showed that, at least for salivary glands, the expression of tight junction proteins including CLDN-4 is similar between swine and humans [53]. Based on the observed diminished CLDN-4 expression on the pOEC membrane and the potential relevance of that expression for tight junction formation, epithelial barrier function, and susceptibility to co-infections, this study also investigated if *Ct* infection leads to a decrease in the overall mRNA, protein, and/or membrane-bound CLDN-4 expression (Aim 3).

To accomplish our three aims, pOECs were infected with *Ct* for up to 72 hpi and at different time points, *Ct*-infected and control cells were harvested to (i) assess *Ct* development via flow cytometry, confocal and transmission electron microscopy (TEM), and qPCR, (ii) study the pOECs immune response to *Ct* infection via NanoString, and (iii) investigate the CLDN-4 mRNA, protein expression, and cellular location via NanoString, flow cytometry, and confocal microscopy, respectively. Our data demonstrate the following: (i) *Ct* developmental cycle in pOECs is productively completed within 48 hpi; (ii) pOECs mainly produce interferon regulated genes and T-cell attracting chemokines; and (iii) while *Ct* infection does not alter the overall mRNA and protein expression of CLDN-4, it leads to a temporary decrease in membrane-bound CLDN-4 with a coinciding increase of CLDN-4 within the *Ct* inclusion. Thereby, we have demonstrated that the *Ct* developmental cycle in pOECs as well as their immune response to infection is highly similar to what has previously been reported for their human cell counterparts [15]. We thereby establish pOECs as a relevant cell model for studies into *Ct* host–pathogen interactions. Furthermore, the *Ct*-induced relocation of CLDN-4 within the infected pOEC justifies the investigation of the underlying molecular mechanisms as well as the downstream effects on tight junction formation, epithelial barrier function, and susceptibility to other pathogens like HIV.

## 2. Results

### 2.1. Chlamydia trachomatis Developmental Cycle in pOECs

**Figure 1:**
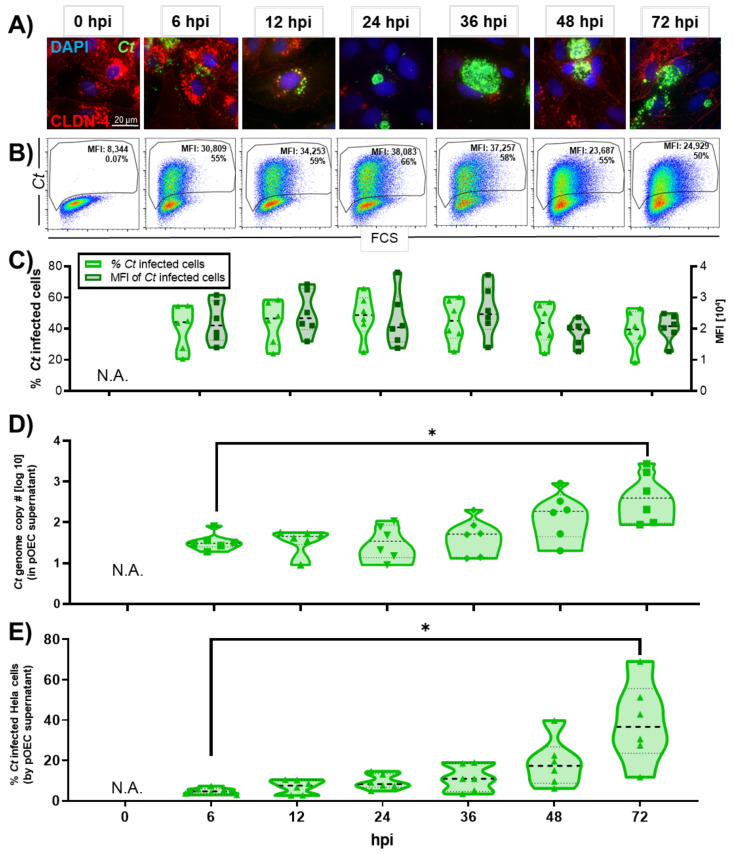
Kinetics of *Chlamydia trachomatis* (*Ct*) infection in porcine oviduct epithelial cells (pOECs). Porcine OECs from six animals were infected with *Ct* (MOI 0.5) for quantification of *Ct* growth in cytoplasmic inclusions and *Ct* excretion into the supernatant was analyzed after 0, 6, 12, 24, 36, 48, and 72 h post-infection (hpi). (**A**) Cells were fixed and permeabilized with ice-cold methanol and stained for fluorescent confocal microscopy: (i) claudin-4 (CLDN-4, red) to visualize the host cell membrane, (ii) 4,6-diamidino-2-phenylindole (DAPI, blue) to show the host cell nucleus, and (iii) an anti-chlamydial LPS antibody (green) to demonstrate *Ct* infection and growth. Representative images from *Ct*-infected pOECs at each time point are shown. (**B**) Cells were also trypsinized, fixed and permeabilized, and stained for *Ct* via indirect immunostaining for flow cytometry. Results show the size of the cells (FSC, x-axis) vs. the *Ct*-content in the cells (*Ct*, y-axis). The percentage of infected cells and the median fluorescence intensity (MFI) of *Ct* are indicated. (**C**) The gate of *Ct* infected cells in panel B was used to calculate the percentage of infected cells (light green, left y-axis) and MFI (dark green, right y-axis). In addition, cell culture supernatants were used to monitor the release of *Ct* particles in two ways: (**D**) *Chlamydia* DNA content in the supernatants was quantified via qPCR (N.D. = not detected); additionally, (**E**) the release of *Ct* particles was also monitored by measuring viable chlamydia particles via flow cytometry after inoculation of HeLa cells with pOEC supernatants. Values for pOEC lines from individual pigs, medians, and 25/75 percentiles are shown. Comparisons were made using one-way ANOVA test and Tukey’s post-test. * *p* < 0.05.

*Chlamydia* infection was first confirmed by fluorescence confocal microscopy. Confocal micrographs showed that *Ct* had entered pOECs by 6 hpi (Figure 1A). At this early stage of infection, the inclusions were small and distributed across the cell cytoplasm. By 24 hpi, inclusion bodies were much larger and localized next to the nucleus. By 36 hpi, the inclusions had further increased in size, taking up most of the host cell (Figure 1A). We also used flow cytometry to corroborate these results (Figure 1B,C). At 6hpi, 44% (median) of pOECs were infected with *Ct* (Figure 1C). The frequency of infected cells stayed relatively constant with a non-significant decline towards the end of this study’s time kinetic. In addition, since pOECs still possessed the ability to proliferate during culture, this frequency might be underestimated. Within infected cells, the median fluorescence intensity (MFI), which indicates the content of *Ct* particles per cell, increased by 21% from 6 to 36 hpi and then declined by 33% from 36 to 72 hpi (Figure 1C). 

In addition to monitoring the *Ct* developmental cycle in the pOECs, we monitored the release of *Ct* from pOECs into the supernatant (Figure 1D,E). First, we extracted DNA from the *Ct*-infected pOECs supernatants and quantified the *Ct* genome copy numbers by qPCR (Figure 1D). While no *Ct* genome copies could be detected prior to inoculation (0 hpi), we detected a median *Ct* genome copy number of 30 with a range from 19 to 81 at 6 hpi. The median *Ct* genome copy numbers were static until 36 hpi. By 48 and 72 hpi, the median *Ct* genome copy numbers increased significantly to 187 and 393, respectively. To corroborate these *Ct* genome copy number data, we used the cell culture supernatants from the *Ct*-infected pOECs to infect HeLa cells; and we measured the *Ct* infection of HeLa cells by flow cytometry (Figure 1E). From 6 to 36 hpi, 5–11% of cells were positive for *Ct*; and at 48 hpi, this percentage increased to 17%. By 72 hpi, the infection rate significantly increased to 37%. These data confirm that *Ct* can infect pOECs and complete its developmental cycle by ~48 hpi: *Ct* infection of pOECs was completed within 6 hpi; by 24 hpi, the *Ct* inclusion had migrated to the cell nucleus; transition from RB to EB occurred as early as 30 hpi; and infectious *Ct* particles were released from pOECs by 48 hpi.

**Figure 2:**
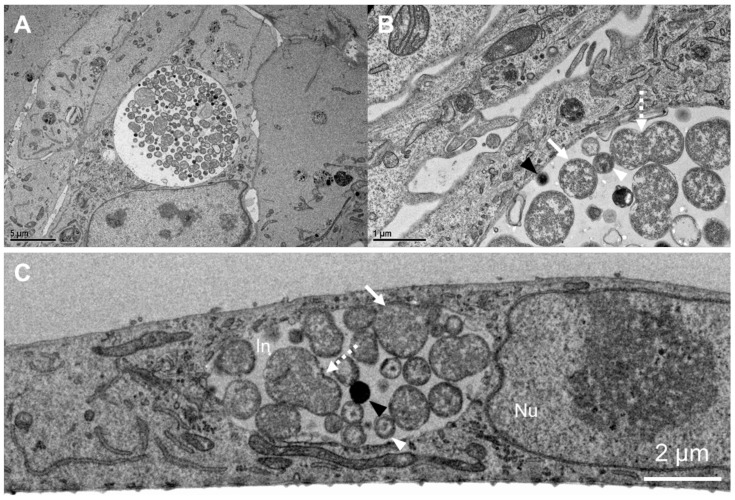
Transmission electron microscopy (TEM) images of *Chlamydia trachomatis* infected porcine oviduct epithelial cells (pOECs). Porcine (OECs) were infected with *Chlamydia trachomatis* (*Ct*) for 30 h and then fixed and prepared for TEM. (**A**) shows an overview of a *Ct*-infected cell with a visible inclusion in the center of the image—5000× magnification. (**B**) shows a 30,000× magnification with visible reticulate bodies (RBs, white arrow), dividing RBs (dashed arrow), intermediate bodies (IBs, white arrow heads), and elementary bodies (EBs, black arrowhead). (**C**) shows a cross-sectional image of the pOECs layer at 8000× magnification with a visible nucleus (Nu), inclusion (In), RBs (arrow), dividing RBs (dashed arrow), IBs (white arrowhead), and EBs (black arrowhead).

To provide further confirmation that *Ct* can efficiently undergo the full developmental cycle within pOECs, TEM images were taken from *Ct* infected pOECs at 30 hpi (Figure 2). These TEM images visualize chlamydial dividing RBs, as well as EB particles: this data further validates that *Ct* can undergo the developmental changes required to successfully complete its infectious cycle in pOECs and also that this cycle follows the 48-h timeline—similar to the *Ct* developmental cycle in human cells [4].

### 2.2. Innate Immune Response of Porcine Oviduct Epithelial Cells to Chlamydia trachomatis Infection

The innate immune response of pOECs to *Ct* infection was analyzed by NanoString mRNA quantification of 31 immune molecules relevant to the human immune response to *Ct* (Table 1).

**Figure 3:**
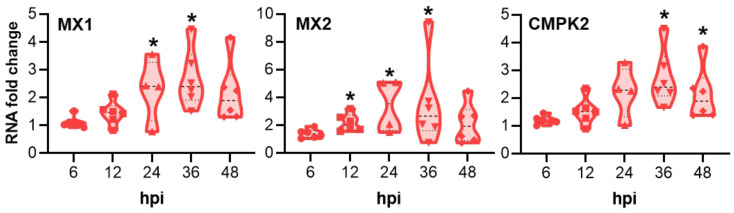
*Chlamydia trachomatis* infection of porcine oviduct epithelial cells (pOECs) induces mRNA fold changes of interferon regulated genes. mRNA fold changes between *Chlamydia trachomatis* infected and MOCK-infected porcine oviduct epithelial cells was calculated via NanoString at 6, 12, 24, 36, and 48 h post infection (hpi). Values for pOEC lines from individual pigs, medians, and 25/75 percentiles are shown. Data comparisons between MOCK-infected and infected cells were performed by the NanoString n solver software using a two-tailed *t*-test. * *p* < 0.05.

**Figure 4:**
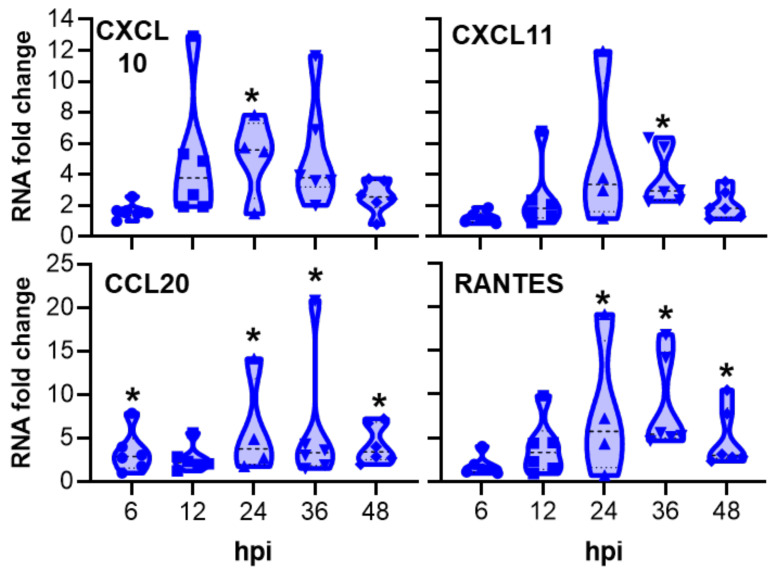
*Chlamydia trachomatis* infection of porcine oviduct epithelial cells induces mRNA fold changes of T-cell attracting chemokines. mRNA fold changes between *Chlamydia trachomatis* infected and MOCK-infected porcine oviduct epithelial cells (pOECs) were calculated via NanoString at 6, 12, 24, 36, and 48 h post infection (hpi). Values for pOEC lines from individual pigs, medians, and 25/75 percentiles are shown. Data comparisons between MOCK-infected and infected cells were performed by the NanoString n solver software using a two-tailed *t*-test. * *p* < 0.05.

*Chlamydia trachomatis* infection induced the highest mRNA upregulation compared with MOCK-infected pOECs at 24 hpi (Table 1), which is also the peak of in vitro infection (Figure 1C). Within the 31 investigated genes, we saw three main groups: (1) 15 targets were not significantly affected; (2) 7 were significantly upregulated at some time points but below a threshold chosen for biological significance of a 2× up- or downregulation; and (3) 9 targets were affected in a significant and biologically relevant manner. Although CCL4 belonged to the third group, this chemokine was excluded from the analysis based on its low gene count in NanoString (maximum of 25 counts at 6 hpi, Supplementary Table S1). Besides an early downregulation of the serum amyloid A2 protein (SAA2), the highest mRNA fold changes were detected for the interferon regulated genes MX1, MX2, and CMPK2, and for the chemokines CCL20, RANTES, CXCL10, and CXCL11 (Figure 3 and Figure 4, respectively).

For the interferon regulated genes, *Ct* infection induced a significant increase in the MX1 and MX2 mRNA fold change (between ~2 and 3.5) at 12, 24 and 36 hpi, while CMPk2 mRNA fold change increased between 36 and 48 hpi to ~2.6 and 2, respectively (Figure 3).

For the chemokines, there was a significant increase in the CXCL10 mRNA fold change at 24 hpi (7.95) and in CXCL11 at 36 hpi (3.45) (Figure 4). In addition, a significant increase in the CCL20 mRNA fold change was observed at 6, 24, 36, and 48 hpi, with the peak at 24 hpi (5.16). *Chlamydia trachomatis* infection also induced a significant increase in RANTES mRNA fold change between 24 hpi and 48 hpi, with the highest fold change at 36hpi (7.42).

### 2.3. Effect of Chlamydia trachomatis Infection on the CLDN-4 mRNA and Protein Expression

During the study of the *Ct* developmental cycle by confocal microscopy, an interesting effect was noticed regarding the expression of the tight junction protein CLDN-4 over the course of the *Ct* infection. As shown in Figure 1A, while CLDN-4 could be used to visualize the cell membrane before and at the very early and late phases of the *Ct* infection process, CLDN-4 was largely absent on the cell membrane of *Ct* infected cells between 12 and 36 hpi—the main phase of *Ct* propagation [4]. This observation led us to ask two questions. (1) Does *Ct* affect the overall CLDN-4 mRNA and/or protein expression in pOECs?; (2) If these overall CLDN-4 expression levels are unaltered, how else can the decrease in membrane-bound CLDN-4 expression be explained?

#### 2.3.1. Effect of *Chlamydia trachomatis* Infection on the Overall CLDN-4 mRNA and Protein Expression

**Figure 5:**
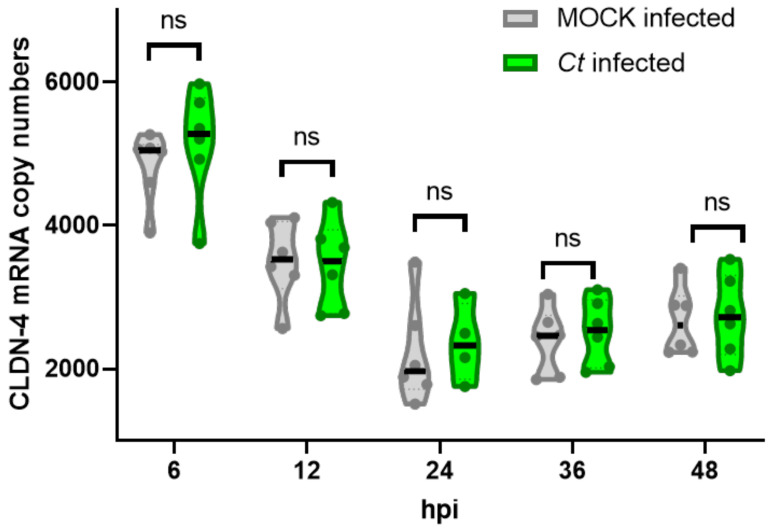
Claudin-4 mRNA expression in porcine oviduct epithelial cells (pOECs) is not affected by *Chlamydia trachomatis*. Porcine oviduct epithelial cells were infected with *Chlamydia trachomatis* (*Ct*) at a multiplicity of infection (MOI) of 0.5 for 0, 6, 12, 24, 36, 48, and 72 h post infection (hpi). Then, RNA was isolated for claudin (CLDN)-4 mRNA quantification via NanoString in MOCK infected cells (grey) or *Ct*-infected cells (green). Violin plots show medians (black line), quartiles (dotted lines) and values from pOECs from individual pigs (round symbols). Statistical analysis was performed via mixed effects model analysis comparing infection-induced CLDN-4 expression changes within time points. No statistically significant changes (*p* < 0.05) were noted.

**Figure 6:**
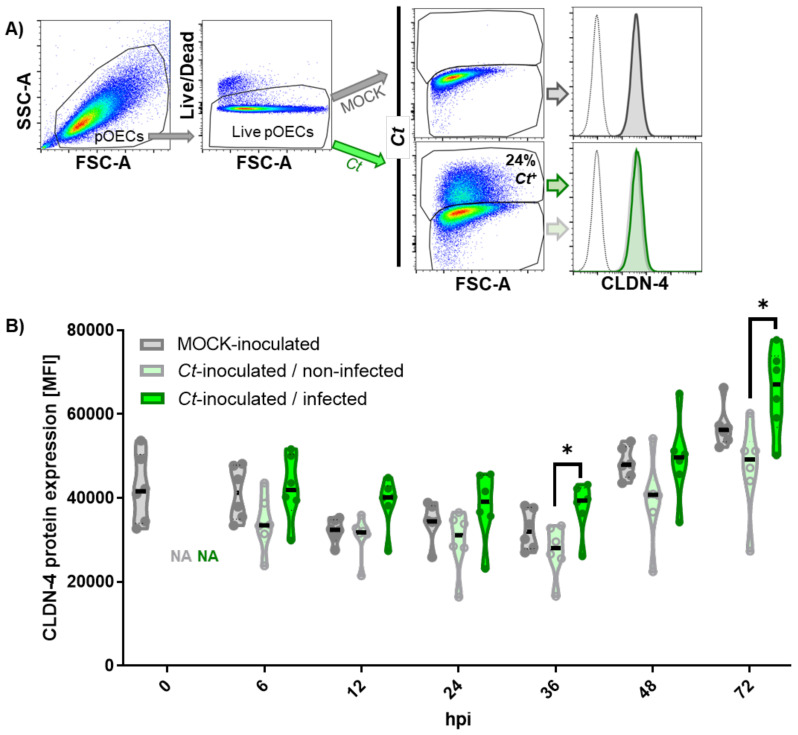
Total Claudin-4 protein expression in porcine oviduct epithelial cells (pOECs) is not decreased by *Chlamydia trachomatis*. Porcine OECs were inoculated with either MOCK or *Chlamydia trachomatis (Ct*, MOI 0.5) for 0, 6, 12, 24, 46, 48, and 72 h post infection (hpi). Post culture, cells were harvested and stained for *Ct* and claudin (CLDN)-4 protein expression analysis via flow cytometry. (**A**) Gating hierarchy for CLDN-4 expression in *Ct*-infected and non-infected pOECs. Porcine OECs were identified by cell size (FSC-A) and granularity (SSC-A). Live pOECs were selected via Live/Dead Near infra-red discrimination staining. Live pOECs of MOCK-inoculated (upper pseudocolor plot) or *Ct*-inoculated (lower pseudocolor plot) were then distinguished into *Ct*-infected and non-infected cells using cell size (FSC-A) and a *Ct*-specific antibody staining (*Ct*). Claudin-4 expression was then analyzed in non-infected cells from MOCK inoculated pOECs (grey, upper histogram), and non-infected cells (grey solid line) and *Ct*-infected cells (green solid line) from *Ct*-inoculated pOECs. (**B**) Violin plots show the median fluorescence intensity (MFI) of CLDN-4 protein expression in MOCK-inoculated pOECs (grey), *Ct*-inoculated/non-infected pOECs (light grey borders with light green filling) and *Ct*-inoculated/infected pOECs (green). Violin plots include medians (black line), quartiles (dotted lines) and MFI values from pOEC lines from individual pigs (round symbols). Statistical analysis was performed for 6–72 hpi time points via 2-way ANOVA with Geisser-Greenhouse correction and Tukey’s multiple comparisons test. * *p* < 0.05.

To answer the first question if *Ct* leads to an overall downregulation of CLDN-4 in infected pOECs, we quantified and compared both the overall mRNA and protein expression levels in *Ct*- and MOCK-infected pOECs via NanoString and flow cytometry (Figure 5 and Figure 6), respectively. While we observed a change over time, the CLDN-4 mRNA copy numbers did not differ significantly between MOCK- and *Ct*-infected pOECs at any analyzed time point (Figure 5).

Similar to the CLDN-4 mRNA results, flow cytometry showed that also the overall CLDN-4 protein expression levels were not altered by *Ct* infection at any studied time point (Figure 6).

Thereby, we could conclude that the diminished membrane-bound CLDN-4 expression in *Ct*-infected pOEC could not be explained by an overall downregulation of CLDN-4 mRNA or protein expression.

#### 2.3.2. *Chlamydia trachomatis* Infection of Porcine Oviduct Epithelial Cells Diminishes the Expression of Claudin-4 on the Host Cell Membrane

**Figure 7:**
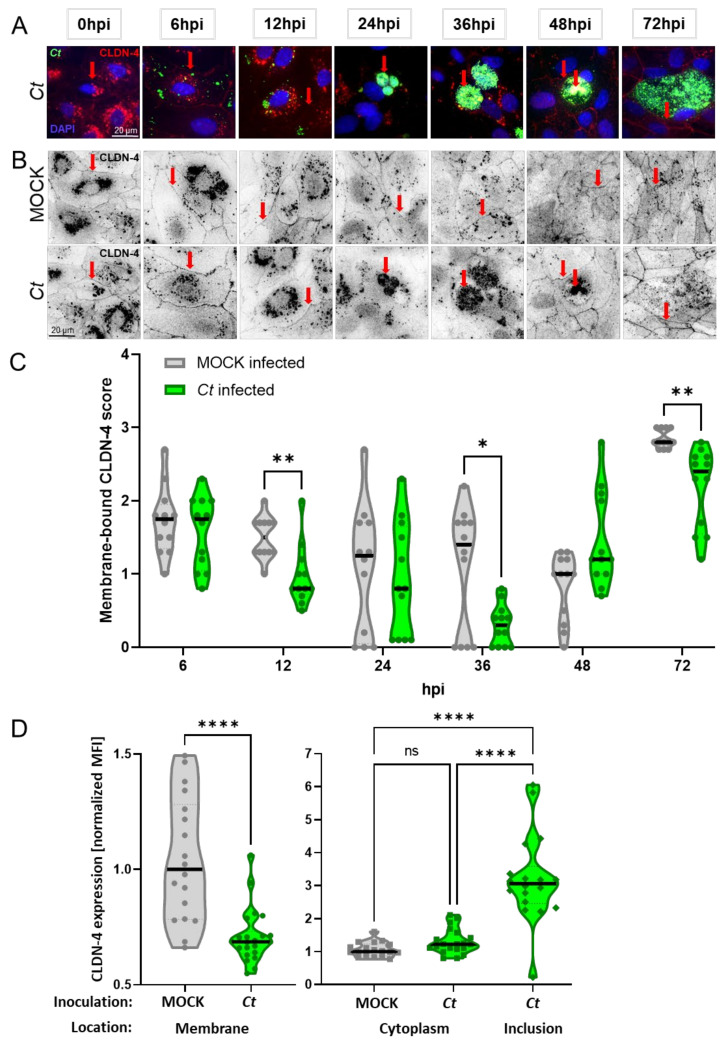
Membrane-bound claudin-4 protein expression in porcine oviduct epithelial cells is decreased during *Chlamydia trachomatis* propagation. Porcine oviduct epithelial cells (pOECs) were cultured on cover slips and infected with *Chlamydia trachomatis* (*Ct*, MOI 0.5) for 0, 6, 12, 24, 36, 48, and 72 h post infection (hpi). Cells were stained for *Ct* (green), DNA (DAPI, blue) and claudin-4 (CLDN-4, red) for confocal microscopy. (**A**) shows an overlay image from *Ct*-infected cells shown in panel A. Red arrows are positioned as the ones in the *Ct*-infected pOECs of panel B: this shall demonstrate the co-localization of CLDN-4 with the cell membrane (0-12 and 48-72 hpi) as well as its co-localization with *Ct* inclusions at 24–48 hpi. (**B**) shows a monochrome CLDN-4 expression in MOCK-infected cells (top row) or *Ct*-infected cells (bottom row). Red arrows in the MOCK-infected cells highlight the membrane-bound CLDN-4 expression throughout the time course of the experiment. For *Ct*-infected pOECs, red arrows are co-localized with red arrows in panel A to better visualize co-localization of CLDN-4 with the cell membrane or *Ct* inclusions. (**C**) Membrane-bound CLDN-4 expression was scored by four independent and blinded evaluators; each evaluator provided a score for MOCK-infected cells (grey) or *Ct*-infected cells (green) ranging from 0–3—0 being absent, 3 being strongest expression. Violin plots show medians (black solid lines), quartiles (dotted lines) and individual data points. These data points show the average evaluator score for each of the pOEC lines from three pigs. This average is based on the scores from 4–10 images per pOEC line from an individual pig and time point. Statistical analysis was performed via 2-way ANOVA with Geisser-Greenhouse correction and Tukey’s multiple comparisons test. * *p* < 0.05, ** *p* < 0.01. (**D**) At 36 hpi, fluorescence signals of CLDN-4 on the pOEC membrane, and within the cytoplasm and the *Ct* inclusion were quantified by ImageJ—for details, see Materials and Methods. The violin plots show the median (black solid lines), quartiles (dotted lines) and individual data points. Statistical analysis was performed using an ordinary one-way ANOVA with Tukey’s multiple comparisons test. * *p* < 0.05, **** *p* < 0.0001.

Since the diminished membrane-bound CLDN-4 expression in *Ct*-infected pOEC could not be explained by an overall downregulation of CLDN-4 mRNA or protein expression, we performed a series of *Ct*-infection time kinetics with a focus on confocal microscopy analysis of the location of CLDN-4 within pOECs: we infected pOEC lines from three pigs with MOCK or *Ct* followed by the same *Ct*, DAPI, and CLDN-4 staining and downstream confocal imaging analysis as used in Figure 1A. The progression of *Ct* infection can be tracked by the increase in the size of *Ct* inclusions (Figure 7A). To further emphasize the location of CLDN-4, black-and-white images of CLDN-4 expression in MOCK- and *Ct*-infected pOECs from a representative pOEC line are shown in Figure 7B. These images demonstrate that while the CLDN-4 expression in MOCK-infected pOECs stays at the cell membrane, CLDN-4 in *Ct*-infected pOECs is present on surface only at the beginning (0 and 6 hpi) and at the end (48 and 72 hpi) of the *Ct* infection cycle. In contrast, at 24 and 36 hpi, CLDN-4 expression was diminished on the surface of *Ct*-infected pOECs. Interestingly, in the same time span, CLDN-4 aggregates became visible within the *Ct* inclusions: this finding suggests that during the time of *Ct* growth, CLDN-4 was relocated into the *Ct* inclusion (Figure 7B). To enable a quantification of the membrane-bound CLDN-4 expression, four independent and blinded investigators scored multiple images of pOEC lines from different pigs and timepoints from these MOCK- and *Ct*-infected pOECs: scores range from 0 being absent to 3 for the highest CLDN-4 expression. The scoring confirmed the initial observation: In the early stages of infection, at 0 and 6 hpi, CLDN-4 expression was strongest at the cell surface in both MOCK- and *Ct*-infected pOECs: the median CLDN-4 expression scores are ~1.8 for both, MOCK- and *Ct*-infected pOECs (Figure 7C). However, thereafter, the membrane-bound CLDN-4 expression was significantly decreased in *Ct*-infected pOECs: at 12 hpi, the median CLDN-4 score for MOCK is with ~1.5 about twice as high as the ~0.8 score for *Ct*-infected pOECs. At 36 hpi, while MOCK-infected pOECs still had a median score of ~1.3, the membrane bound CLDN-4 expression in *Ct*-infected pOECs was nearly absent – median score of ~0.3. At 48 hpi, the membrane-bound CLDN-4 expression in *Ct*-infected pOECs returned to levels comparable to the MOCK-infected pOECs – median scores ~1. At 72 hpi, both MOCK- and *Ct*-infected pOECs showed a strong CLDN-4 expression on the cell surface. To further validate the decrease in membrane-bound CLDN-4 expression and to study CLDN-4 in other cell compartments, CLDN-4 expression on the cell membrane, in the cell cytosol, and in the *Ct* inclusion was quantified via Image J at 36 hpi, the time point with the strongest reduction in CLDN-4 expression (Figure 7D). This analysis not only confirmed that membrane-bound expression of CLDN-4 was significantly reduced in *Ct*-infected pOECs but it also showed that while there was no difference in the cytosolic CLDN-4 expression between MOCK- and *Ct*-infected pOECs, the CLDN-4 expression within the *Ct* inclusions, was three times increased compared to the cytosolic expression of both MOCK- and *Ct*-infected cells (Figure 7D). Together, these observations confirm the reduction in membrane-bound CLDN-4 expression during the *Ct* propagation phase between 12 and 36 hpi with an increased presence of CLDN-4 within the *Ct* inclusion (Figure 7).

Thereby, we could conclude that while the overall CLDN-4 mRNA and protein expression in pOECs is unaltered (Figure 5 and Figure 6), *Ct* infection does lead to a timely defined relocation of the tight junction protein CLDN-4 from the cell membrane into the *Ct* inclusion (Figure 7).

In summary, our data not only show that *Ct* can successfully complete its cell cycle in pOECs (Figure 1 and Figure 2), but this infection also induces an innate immune response similar to their human counterparts (Table 1, Figure 3 and Figure 4, and Supplementary Table S1). We additionally use these relevant primary pOECs to demonstrate that while the overall mRNA and protein expression of CLDN-4 is unaltered (Figure 5 and Figure 6, respectively), *Ct* infection triggers a redirection of CLDN-4 into the *Ct* inclusion that leads to a decrease in CLDN-4 expression on the pOEC membrane (Figure 7).

## 3. Discussion

While *Ct* infection in porcine genital tract epithelial cells has been previously described [42], this study provides a detailed analysis of the developmental cycle of *Ct* in pOECs—the counterpart of human Fallopian tube epithelial cells. To facilitate the utilization of the relevant swine model for *Ct* host–pathogen interactions, we characterized the developmental cycle of *Ct* in pOECs, the induced cellular immune response, and the effect of *Ct* infection on the pOEC expression of the tight junction protein CLDN-4.

### 3.1. Chlamydia trachomatis Developmental Cycle in Porcine Oviduct Epithelial Cells

Using confocal microscopy and flow cytometry, we demonstrate that *Ct* can complete its developmental cycle in pOECs: Infection takes place within the first six hours of inoculation, chlamydial load peaks ~24 hpi and while the inclusion continues to grow until 72 hpi, chlamydial load decreases at the later time points probably due to the extrusion of chlamydial EBs (Figure 1A–C). This timeline is in accordance with the developmental cycle of *Ct* in human cells [4,12,13,14]. We furthermore validate a successful *Ct* infection in pOECs and transition from chlamydial RBs to EBs: at 30 hpi, TEM images show chlamydial inclusions within pOECs including RB and EB particles. On top, we identified dividing RBs with daughter cells of asymmetric and symmetric volume; these division mechanisms mimic both *Ct* propagation mechanisms—“budding” [54,55,56] and “binary fission” [14] divisions, respectively. This timeline further supports not only the data of Guseva et al. showing chlamydial RBs and EBs in other porcine genital tract epithelial cells [42] but also the general timeline for the RB to EB transition of *Ct* [4].

To ensure that the completion of the *Ct* developmental cycle leads to the release of infectious chlamydial EBs, we analyzed the presence of *Ct* in the supernatants of these cultures not only by qPCR (Figure 1D) but also by using these supernatants to infect HeLa cells (Figure 1E). Our results show that the *Ct* genome copy numbers and infectious *Ct* particles (EBs) increased by number at 48 hpi and significantly at 72 hpi: Therefore, *Ct* EBs were released at ~48 hpi; this timeline once more follows the timeline described for *Ct* infection of human cells [4,12,13,14].

In summary, our results demonstrate that *Ct* completes its developmental cycle in pOECs with the release of infectious EBs within 48 h. This timeline corresponds to the developmental cycle of *Ct* in human cells [10,11]; and it supports the use of the pOECs to study *Ct* infection and the involved complex interactions between *Ct* and its host cell.

### 3.2. Innate Immune Response of Porcine Oviduct Epithelial Cells to Chlamydia trachomatis Infection

The innate immune response of pOECs to *Ct* infection was analyzed via NanoString in a time kinetic spanning the *Ct* infection cycle: mRNA expression of 31 genes related with *Ct* infection were compared between MOCK- and *Ct*-infected pOECs (Table 1, Figure 3 and Figure 4, and Supplementary Table S1).

Based on the central role of pro-inflammatory cytokines in the anti-*Ct* response, we hypothesized that *Ct* infection would increase the expression of pro-inflammatory cytokines such as IL-6, IL-8, and TNF-α. Yet, surprisingly, none of these cytokines were upregulated to a level that crossed the biological relevance threshold of 1.5× and significance level of *p* < 0.05 (Table 1). While this lack in upregulation was unexpected, these same cytokines were also not upregulated by *Ct* infection of primary human Fallopian tube epithelial cells [15]. Therefore, at least in vitro, genital tract epithelial cells might not be a primary source of pro-inflammatory cytokines in *Ct* infection.

While these pro-inflammatory cytokines were not increased, *Ct* infection of pOECs did lead to a significant increase in the mRNA expression of the interferon regulated genes MX1, MX2, and CMPK2 (Figure 3). As in our study, in vitro studies using murine genital tract epithelial cells have shown that *C. muridarum* infection upregulates the mRNA expression of MX1 and MX2 [57]. MX proteins are GTPases found in almost all vertebrates and best known for their anti-viral activities [58]. Multiple studies have demonstrated an effect of MX protein in viral replication by interference with transcription and translation [58]. In addition, human studies have indicated induction of MX1 and MX2 in ocular *Ct* infection [59]. Based on the role in transcriptional and translational interference, MX1 and MX2 upregulation by pOECs could aim to limit *Ct* propagation and thereby *Ct* infection within the genital tract. Regarding CMPK2, studies using murine genital tract epithelial cells have also shown an upregulation of CMPK2 mRNA upon *C. muridarum* infection [60]. The enzyme CMPK2 not only supplies deoxyribonucleotides for the synthesis of mitochondrial DNA (mtDNA) [61] but it also possesses immunomodulatory and antiviral activities: CMPK2 was shown to limit dengue virus infection of murine and human cells in an IFN-dependent and IFN-independent manner [62]. Taken together, the *Ct*-induced upregulation of interferon regulated genes in pOECs potentially not only limits *Ct* propagation within the infected cell, but it could also promote immunomodulation in the genital tract.

*Chlamydia* infection also induced a significant mRNA upregulation of the chemokines CXCL10, CXCL11, CCL20, and RANTES (Table 1 and Figure 4). The CXCL10, CCL20, and CXCL11 results are in accordance with murine data presented by Rank et al. [16]: they demonstrated that endocervical infection with *C. muridarum* induces an upregulation of CXCL10, CXCL11, and CCL20 mRNA in the mouse cervix at 24 hpi. The receptors to each of these chemokines are expressed on T cells: CXCL10 and CXCL11 are part of a family of high homology chemokines that bind to the CXCR3 chemokine receptor; in contrast, CCL20 binds to the receptor CCR6 [63,64]. In addition, the receptor for the chemokine RANTES is also expressed on T cells—CCR5 [65]. With RANTES, CXCL10, and CXCL11, most of these targets have also been upregulated in apical secretions of primary human Fallopian epithelial cells 48 h post *Ct* infection [15]; and CXCL10 and CXCL11 have also been associated with the increased risk of *Ct* reinfection in women [66]. In addition, using murine genital mucosa, Olive et al. showed that CXCR3 and CCR5 are both required for T-cell mediated protection against *Ct* [67].

In summary, all chemokines upregulated in *Ct*-infected pOECs attract T cells to the site of infection. Based on the central role for T cells and especially IFN-γ producing tissue-homing CD4 T cells, we can conclude that pOECs also play a crucial role in the induction of an anti-*Ct* immune response as well as the generation of anti-*Ct* immunity [19,68,69].

### 3.3. Chlamydia trachomatis Infection of Porcine Oviduct Epithelial Cells Affects Their Claudin-4 and Tight Junction Expression

As mentioned above, during our initial studies on the *Ct* developmental cycle, membrane-bound CLDN-4 expression was diminished during the peak *Ct* propagation—Figure 1A. Claudins are an important structural component of the tight junctions and play a key role in the permeability of epithelial cell layers [45]. A literature search on the effect of *Ct* infection on the expression of the tight junction molecule CLDN-4 and its downstream effects on tight junction formation and the epithelial barrier function revealed three important findings: first, in the mouse model, *C. muridarum* infection has shown to alter the composition of tight junction proteins such as claudins 1-4 in murine oviduct epithelial cells, the counterpart of human Fallopian tube epithelial cells [47]; second, Prozialeck et al. [70] showed that infection of primary human cervical epithelial cells with *Ct* for 24 h disturbed the cell–cell contacts: these cells not only showed a loss of the cell membrane protein N-cadherin but also of their cell–cell adherence junctions [71]; and third, using the human uterine epithelial cell line ECC-1, Mukura et al. showed that *Ct* infection can decrease the CLDN-4 mRNA expression as well as the trans-epithelial electrical resistance (TEER) [48].

Based on this observation and the relevance of CLDN-4, we first tested the hypothesis that *Ct* downregulates the overall mRNA (Figure 5) and protein (Figure 6) expression of CLDN-4 in pOECs. However, in contrast to the finding of Mukura et al. [48], *Ct* did not alter the mRNA expression of CLDN-4 in pOECs (Figure 5). A more recent study by Kumar et al. indicate that *Cm* infection rather increases the mRNA expression of CLDN-4 in infected murine oviduct epithelial (OE) cells; however, this study does not show a timeline of MOCK and *Cm* infected OE cells [47]. In addition, our study furthermore showed that *Ct* also did not alter the overall CLDN-4 protein expression (Figure 6). When comparing MOCK-infected and *Ct*-infected pOECs within *Ct*-inoculated wells, it rather seemed that *Ct*-infected pOECs even had an increased overall protein expression of CLDN-4 (Figure 6). Therefore, we had to reject this hypothesis and find another explanation for the diminished CLDN-4 expression on the pOEC membrane. Since the overall CLDN-4 expression was unaltered, we next hypothesized that *Ct* alters the location of CLDN-4 within infected pOECs. To test this hypothesis, CLDN-4 location was studied via confocal microscopy and the membrane-bound CLDN-4 expression scored over the course of the *Ct* infection and quantified in detail at the peak of the *Ct* effect on CLDN-4 (Figure 7). This analysis not only confirmed that membrane-bound CLDN-4 expression was reduced during the main phase of *Ct* propagation (12–36 hpi) but it also showed that during these times, CLDN-4 was mainly seen within the *Ct* inclusion. Therefore, we could accept our second hypothesis that *Ct* alters the location of CLDN-4 within infected pOECs. We propose the following hypothesis for the loss of membrane-bound CLDN-4: CLDN-4 is constantly internalized and replaced by newly synthesized CLDN-4; however, as *Ct* enhances the fusion of the inclusion with exocytic vesicles, the internalized CLDN-4 cannot be efficiently replaced; consequently, this would lead to a net loss in membrane-bound CLDN-4. Based on the numerous interaction partners during CLDN-4 trafficking, further research will be required to identify the way how *Ct* accomplishes this CLDN-4 relocation to the inclusion. Since this effect was mostly visible during the active propagation phase of *Ct*, we assume that the internalized CLDN-4 is part of the membrane that is used to promote the growth and propagation of chlamydial RBs. To further improve the toolbox for *Ct* pathogenesis [71], we propose that future studies using pOECs should determine the molecular mechanisms by which *Ct* leads to the relocation of CLDN-4 into the *Ct* inclusion.

In addition, studies using pOECs should also analyze how the described CLDN-4 relocation affects both pOEC tight junction expression as well as its impact on the trans-epithelial resistance. Our preliminary TEM analysis of the tight junction formation indicates that compared to MOCK-infected cells, *Ct*-infected pOECs formed less dense cell-to-cell contacts at 30 hpi including a decrease in tight junction expression (Supplementary Figure S1). While these data are not sufficient to draw the conclusion that *Ct* possesses the ability to decrease the expression of pOEC tight junctions, they support future research into the effect of *Ct* infection on tight junction expression in genital tract epithelial cells. However, while the utilized 2D cell culture system allowed a quantification of the CLDN-4 expression in pOECs, the quantification of tight junctions via TEM was more challenging. Therefore, we propose that future studies should use a polarized 3D culture technique as developed by the Nagarajan group [15]: it allows both, not only an optimized TEM quantification of tight junctions, but also TEER analysis. This technique could thereby validate if *Ct* does impair not only the expression of membrane-bound CLDN-4 on pOECs but also of their tight junction expression and epithelial barrier function. These future studies could thereby provide the underlying mechanisms in the increased risk of HIV infection in *Ct* patients [49,50,51,52].

In conclusion, our results demonstrate three important findings: First, *Ct* can complete its developmental cycle in pOECs with the release of infectious EBs. Second, both the *Ct* developmental cycle and innate immune response in pOECs resemble their counterparts in their human counterparts—Fallopian tube epithelial cells. Based on these similarities, primary pOECs represent an excellent model to study the pathogenesis and innate immune response of genital *Ct* infections. And third, during the main propagation phase, *Ct* leads to an accumulation of CLDN-4 within the inclusion that coincides with a decreased CLDN-4 expression on the host cell membrane. This observation justifies future studies into the molecular mechanism of this process as well as its downstream effects on tight junction formation, epithelial barrier function and the increased risk susceptibility to co-infections such as HIV.

## 4. Materials and Methods

### 4.1. Porcine Oviduct Epithelial Cells Isolation and Culture

For pOECs isolation, porcine oviducts were collected from six sows at a local slaughterhouse (City Packing/Neese’s Sausage Co., Burlington, NC, USA) immediately after slaughter. The cell isolation procedure and culture followed a well-established protocol that results in the isolation of human Fallopian tube epithelial cells as described by McQueen et al., 2020 [15]. Oviducts were washed with phosphate-buffered saline (PBS, Corning, Corning, NY, USA) containing antibiotic-antimycotic (anti-anti, Corning), opened to expose the epithelial layer and cut in pieces of about 1 cm. Oviduct pieces were then added into a tube with 20 mL of cell isolation media composed of DMEM/F-12 media (Corning), 1x anti-anti, dispase (*v*/*v* 1:200, Corning), and pancreatin (*v/w* 1:83, MP Biomedicals, Irvine, CA, USA). The tissue was incubated at 4 °C overnight with shaking.

After incubation, the lumen of the oviduct pieces was gently scraped with a scalpel blade to isolate the epithelial cells. Scraped cells and isolation media was collected into a tube and the solution was neutralized with fetal bovine serum (FBS, *v*/*v* 1:2, VWR, Radnor, PA, USA). Cells were then centrifuged at 600× *g* for 5 min at 4 °C and pellet was resuspended with acutase (*v*/*v* 1:3, Corning). The cell solution was then incubated at 37 °C for a total of 30 min, with agitation after 15 min. Cells were centrifuged at 600× *g* for 5 min at 4 °C and pellet was resuspended in 20 mL of “pOEC media” composed of DMEM/F-12 media, Antibiotic-Antimycotic (ThermoFisher, Waltham, MA, USA), 5% FBS, 0.0025% epidermal growth factor (EGF, Corning), and 0.1% insulin, transferrin, selenium (ITS, Corning).

Cell suspension was then seeded in T-75 flasks (Sarstedt, Nümbrecht, Germany) at the density of 8 × 10^6^ cells/flask and incubated at 37 °C until about 70–80% confluency (about 48 h). After incubation, cells were washed twice with PBS and trypsinized with 0.25% trypsin (Corning) for about 15 min at 37 °C. Trypsinization was stopped with pOEC media. Cells were transferred to a tube and centrifuged at 500× *g* for 8 min at 4 °C. Next, cell pellet was resuspended in freezing media composed of 50% DMEM/F-12 media, 40% FBS, and 10% dimethyl sulfoxide (DMSO, Corning), and stored in liquid nitrogen.

### 4.2. Chlamydia trachomatis

The *Ct* serovar E prototype reference strain Bour (ATCC VR-348B, [72,73]) was propagated in HeLa cells using standard technique [74] and purified as previously described [41]. Bacteria were titrated on HeLa cells as previously described [75].

### 4.3. Chlamydia trachomatis Infection of Porcine Oviduct Epithelial Cells

For pOECs culture, frozen pOECs were thawed, washed with DMEM/F-12 media, resuspended in “infection media”: DMEM/F-12 media, 10 μg/mL gentamicin, 5% FBS, 0.0025% EGF, and 0.1% ITS. Then, pOECs were seeded in 24-well plates in the presence or absence of poly-D-lysine coated coverslips at the density of 2 × 10^5^ cells/well. The coverslips were used to facilitate confocal microscopy analysis of *Ct* infection in pOECs. Cells were then incubated at 37 °C until about 70–80% confluency (about 24 h). After pOEC culture in 24-well plates for about 24 h, pOECs were infected with *Ct* (MOI 0.5) for 6, 12, 24, 30, 36, 48, and 72 hpi. For each time point, MOCK-infected pOECs were used as controls: these cells were undergoing the same infection procedure as *Ct*-infected cells but in the absence of *Ct*. After addition of a *Ct* or control solution, the 24-well plates were centrifuged at 37 °C and 900× *g* for 1 h followed by an additional hour of incubation in the incubator. Thereafter, the *Ct* and control solutions were taken off, pOECs washed with culture medium and then incubated for the time of infection in fresh culture medium. Cell culture supernatants were collected at each time point and stored at −20 °C until used to determine the release of *Ct* particles via qPCR.

### 4.4. Fluorescence Confocal Microscopy

*Chlamydia trachomatis* infection in pOECs from six individual animals was evaluated via fluorescence confocal microscopy as previously described [41]. Cell membrane bound CLDN-4 protein expression in pOECs from three individual animals was also evaluated during *Ct* infection also using fluorescence confocal microscopy. Briefly, cells were washed with PBS, fixed and permeabilized with methanol (Millipore-Sigma, Burlington, MA, USA), and first stained with the primary antibodies anti-*Chlamydia* antibody clone ACI (LSBio, Seattle, WA, USA) and anti-CLDN-4 (Abcam, Cambridge, UK) followed by a staining with the secondary antibodies anti-mouse IgG3-Alexa 488 (Southern Biotech, Birmingham, AL, USA) and anti-rabbit polyclonal-Alexa 555 (Southern Biotech) as well as 4′,6-diamidino-2-phenylindole (DAPI) (Tocris, Bristol, UK). Fluorescence images were acquired with a Nikon Eclipse Ti microscope equipped with a 100×/numerical aperture (NA) 1.49 HP Apo TIRF objective (Nikon, Tokyo, Japan), a CSU-X1 (Yokogawa, Tokyo, Japan) confocal spinning-disk system, 405/488/561/647 nm solid state lasers, and an electron-multiplying cooled charge-coupled device camera (EMCCD IXon 897, Andor Technology, Belfast, UK). The Nikon Element software was used for acquisition.

For the CLDN-4 analysis, image stacks were summed, and brightness/contrast was adjusted to a minimum of 9493 and a maximum of 87,853.2 using ImageJ [76]. Thereafter, a total of 215 images from pOECs from three individual swine were evaluated by four independent investigators by giving a score to the CLDN-4 staining ranging from 0 to 3: 0 represents the absence of membrane-bound CLDN-4; 3 shows the strongest membrane CLDN-4 staining.

### 4.5. Flow Cytometry

*Chlamydia trachomatis* developmental cycle in pOECs from six individual animals was also monitored via flow cytometry as previously described [41]. In addition, the CLDN-4 protein expression was quantified in *Ct*- or MOCK-infected pOECs. These pOECs were washed with PBS, trypsinized and stained with Live/Dead Near infra-red. Next, cells were fixed and permeabilized with the eBioscience™ Foxp3/Transcription Factor Staining Buffer Set (ThermoFisher). Then, cells were stained with the primary antibodies anti-*Chlamydia* antibody clone ACI (mouse IgG3, LSBio) and anti-CLDN-4 antibody (rabbit polyclonal, Abcam), and then with the secondary antibodies anti-mouse IgG3-Alexa 488 antibody (Southern Biotech) and anti-rabbit-Alexa 647 (Southern Biotech). Cells were recorded on a Cytoflex flow cytometer using the CytExpert software (Beckman Coulter, Brea, CA, USA). Data analysis was performed with FlowJo version 10.5.3 (FLOWJO LLC, Ashland, OR, USA). The gating hierarchy used for the differentiation of *Ct*-infected and MOCK-infected cells is shown in Supplementary Figure S2. The *Ct* gate was set based on MOCK-infected control cells.

### 4.6. Detection of Chlamydia via qPCR

The DNA from pOECs supernatants from six individual animals was used to measure the *Chlamydia* shedding via Taqman qPCR assay. Prior to performing the qPCR, DNA was isolated from pOEC supernatants as previously described [41]. Primers and probe targeting the chlamydial 23S rRNA used for *Chlamydia* quantification were previously described [41]: (i) Fwd primer: CCTAAGTTGAGGCGTAACTG, (ii) Rv primer: GCCTACTAACCGTTCTCATC, (iii) Probe: FAM-TTAAGCACGCGGACGATTGGAAGA-TAMRA. A total of 5 µL DNA was mixed to 7.5 µL of KAPA PROBE Fast qPCR Master (KAPA Biosystems, Wilmington, MA), 0.3 µL of each primer, 0.3 µL of probe (10 µM), and 1.6 µL of nuclease free water. qPCR conditions were as follows: 95 °C for 3 min, followed by 40 cycles with denaturation at 95 °C for 10 s and annealing/elongation for 30 s at 60 °C. The Taqman qPCR was run on a qTOWER3G qPCR machine (Analytik Jena, Jena, Germany). A standard curve of *Chlamydia* gBlocks (IDT^®^ Integrated DNA Technologies, Newark, NJ, USA) was included on every plate to determine the number of *Chlamydia* particles.

### 4.7. NanoString

The innate immune response and CLDN-4 expression in MOCK- and *Ct*-infected pOECs from six individual animals was monitored using NanoString gene expression analysis (NanoString Technologies, Inc., Seattle, WA, USA). At each time point, pOECs were washed with 500 µL of 1× PBS/well and 500 µL of Trizol (TRIzol LS Reagent, Thermo Fisher) was added into each well. Total RNA isolation using Trizol was preformed according to the manufacturer’s instructions. RNA was eluted in 20 μL nuclease-free water and RNA purity and quantity were determined using a Nanodrop 1000 (Thermo Fisher).

The mRNA expression of immune parameters and CLDN-4 was then measured via NanoString nCounter platform by calculating the mRNA fold change between *Ct* infected and MOCK-infected pOECs. mRNA input was normalized based on Nanodrop value to 100 ng and processed according to the nCounter MAX/FLEX System User Manual (NanoString Inc., MAN-C0035-07). Briefly, normalized mRNA was hybridized with biotin-labeled capture probes and fluorescently labeled reporter probes for 19 h at 65 °C. Following hybridization, samples were transferred into a NanoString cartridge and loaded onto the nCounter Prep Station instrument set on high sensitivity. Next, cartridge was transferred into the nCounter Digital Analyzer instrument, where excess capture probe and reporter probe were removed, and hybridized mRNAs were immobilized for imaging (555 fields of view). The panel of immune genes investigated is listed in Table 1. Following image acquisition, mRNA counts were analyzed using nSolver analysis software.

### 4.8. Statistical Analysis

For the NanoString gene expression analysis, data was analyzed by NanoString n solver (nSolver 4.0 Analysis Software, Inc., Seattle, WA, USA), which make comparisons between MOCK- and *Ct*-infected pOECs using a two-tailed *t* test. For the rest of the data, statistical analyses were performed by GraphPad Prism (GraphPad 8.0 Software, Inc., La Jolla, CA, USA): Depending on the dataset, either a mixed model or a repeated-measures one- or two-way ANOVA were performed; post hoc multiple comparisons were performed either using Šídák’s or Tukey’s test. The usage of each statistical analysis is reported in the figure legends. Differences were defined significant for *p* < 0.05.

## Figures and Tables

**Table 1:** Gene regulation induced by *Ct* infection in porcine oviduct epithelial cells (pOECs). mRNA fold changes between *Ct* infected and MOCK-infected pOEC lines from six individual pigs was calculated via NanoString at 6, 12, 24, 36, and 48 h post infection (hpi). Data were analyzed by NanoString n solver software by comparing gene counts between neg and pos samples including the integrated statistical analysis: grey cells indicate significant differences with *p* < 0.05.

Gene	6 hpi	12 hpi	24 hpi	36 hpi	48 hpi	*Average*
ADAMTS9	1.83	1.42	1.73	1.12	−1.37	*0.95*
CCL20	2.8	2.43	5.16	3.83	3.82	*3.61*
CCL4	5.49	3.07	2.91	1.86	2.53	*3.17*
CMPK2	1.2	1.47	2.32	2.59	1.98	*1.91*
CSF3	1.6	1.93	1.64	1.37	1.26	*1.56*
CXCL10	1.59	3.86	7.95	4.5	2.3	*4.04*
CXCL11	1.18	1.97	4.81	3.45	1.9	*2.66*
CXCL8	1.18	−1.01	1.12	1.5	1.99	*0.96*
CXCL9	−1.15	−1.28	1.11	−1.2	1.02	*−0.3*
EBI3	−1.08	−1.23	1.36	1.12	1.14	*0.26*
Flt-3L	−1.01	1.14	1.04	1.14	1.28	*0.72*
HERC5	1.09	1.35	1.89	1.93	1.7	*1.59*
ICAM1	1.28	1.23	1.3	1.36	1.2	*1.27*
IL-1 alpha	1.36	1.21	1.24	1.18	1.44	*1.29*
IL-10	−1.23	1.03	−1.41	−1.29	−1.39	*−0.86*
IL-15	1.06	1.52	1.6	1.39	1.11	*1.34*
IL-16	−1.03	1.22	−1.02	1.14	1.05	*0.27*
IL-1RA	1.02	1.39	1.49	1.03	1.29	*1.24*
IL-6	1.28	1.26	1.7	1.58	1.49	*1.46*
IL-7	1.17	1.21	1.85	1.84	1.37	*1.49*
MMP-2	1.06	−1.11	1.3	1.18	−1.03	*0.28*
MMP-9	1.23	1.09	−1.11	1.09	1.43	*0.75*
MX1	1.11	1.39	2.31	2.53	1.98	*1.86*
MX2	1.38	2.12	3.49	2.66	1.74	*2.28*
NEURL3	−1.06	−1.19	−1.08	−1.36	1.12	*−0.71*
OAS2	1.11	1.3	1.92	2.4	1.84	*1.71*
RANTES	1.6	2.88	5.42	7.42	4.08	*4.28*
SAA2	−2.55	1.08	−1.2	−1.39	1.02	*−0.61*
TGF-b	1.05	−1.08	1.09	−1.03	−1.14	*−0.22*
TNF-alpha	−1.13	−1.16	1.73	−1.23	−1.42	*−0.64*
VEGF	1.08	1.06	1.21	1	−1.04	*0.66*
***Average***	*0.8*	*0.95*	*1.75*	*1.43*	*1.15*	

## Data Availability

The data presented in this study are available on request from the corresponding author.

## Supplementary Materials

The following are available online at https://www.mdpi.com/article/10.3390/pathogens10101270/s1, Supplementary Table S1: *Chlamydia* immune related gene count and fold change in porcine oviduct epithelial cells. Supplementary Figure S1: Effects of *C. trachomatis* infection on porcine oviduct epithelial cell-to-cell contacts. Supplementary Figure S2: Gating hierarchy for CLDN-4 protein expression during *Ct* infection via flow cytometry.
