# Supplementary material for: Host–Pathogen Interactions of Chlamydia trachomatis in Porcine Oviduct Epithelial Cells
Source: Pathogens. 2021 Oct 1;10(10):1270. doi: 10.3390/pathogens10101270 (PMC8540921; doi:10.3390/pathogens10101270)
Supplement: Supplementary file 1 [file pathogens-10-01270-s001.zip › supple/Ct HPI in pOEC_Supplementary Figure S1.pdf]

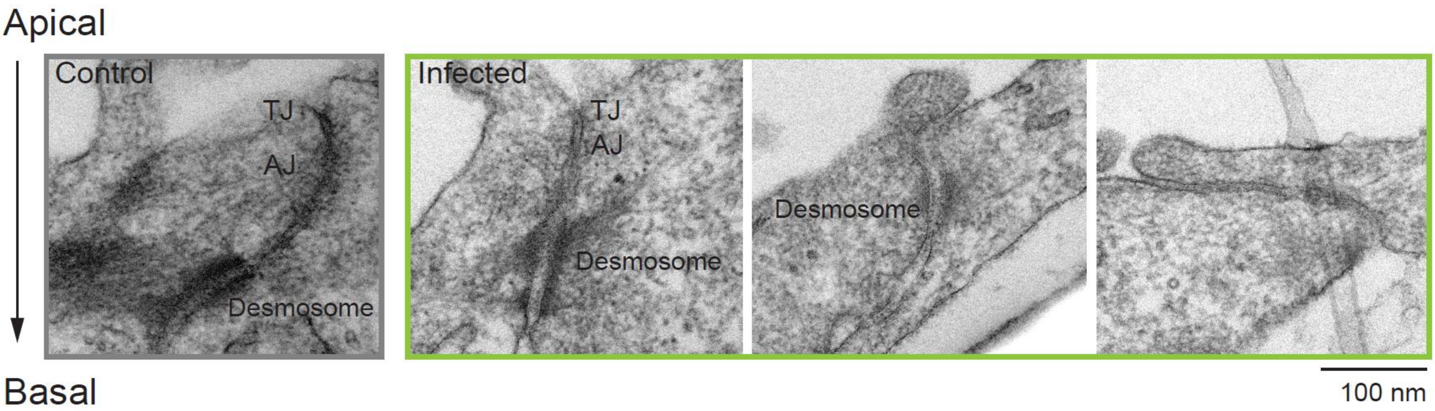

Supplementary Figure S1. Effects of *C. trachomatis* infection on porcine oviduct epithelial cell-to-cell contacts. Transmission electron microscopy (TEM) was performed on porcine oviduct epithelial cells (pOECs) either MOCK (Control, left image) or *Ct*-infected (green frame) at 30 hours post infection. The representative TEM images show tight junctions (TJ), adherence junctions (AJ) and desmosomes. In contrast to the strong cell-to-cell contacts in control cells, cell-to-cell contacts in *Ct*-infected cells appear less dense.
