# Supplementary material for: Host–Pathogen Interactions of Chlamydia trachomatis in Porcine Oviduct Epithelial Cells
Source: Pathogens. 2021 Oct 1;10(10):1270. doi: 10.3390/pathogens10101270 (PMC8540921; doi:10.3390/pathogens10101270)
Supplement: Supplementary file 1 [file pathogens-10-01270-s001.zip › supple/Ct HPI in pOEC_Supplementary Figure S2.pdf]

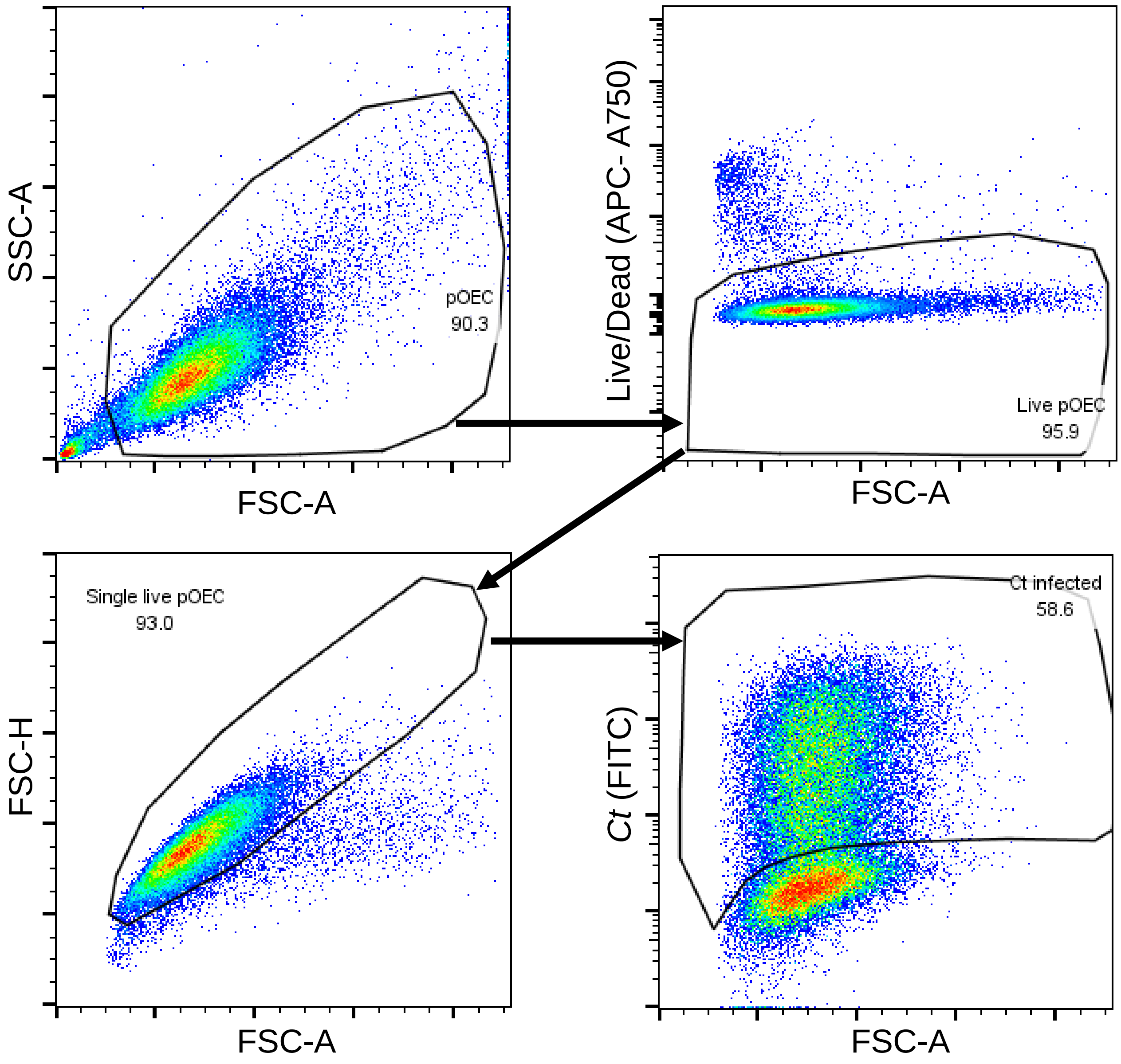

Supplementary Figure S2. Gating hierarchy for *Chlamydia trachomatis* (*Ct*) infection analysis via flow cytometry. Porcine oviduct epithelial cells (pOEC) were infected with *Ct* (MOI 0.5) for 0, 6, 12, 24, 36, 48, and 72 hours post infection. Cells were trypsinized, fixed and permeabilized, and stained for *Ct* via indirect immunostaining for flow cytometry. pOEC were identified by cell size (FSC-A) and granularity (SSC-A). Live cells were then identified by a gate on Live/Dead Near infra-red staining. Next, doublets were excluded by an FSC-A (area)/FSC-H (height) gate. *Ct* infected pOEC were identified via FITC-conjugated anti-chlamydial LPS antibody. Gating is based on non-infected control cells.
