# Supplementary material for: Host–Pathogen Interactions of Chlamydia trachomatis in Porcine Oviduct Epithelial Cells
Source: Pathogens. 2021 Oct 1;10(10):1270. doi: 10.3390/pathogens10101270 (PMC8540921; doi:10.3390/pathogens10101270)
Supplement: Supplementary file 1 [file pathogens-10-01270-s001.zip › supple/Ct HPI in pOEC_Supplementary Table S1.pdf]

Supplementary Table S1. Chlamydia immune related gene count and fold change in porcine oviduct epithelial cells. mRNA fold change between *Chlamydia trachomatis* infected (pos) and non-infected (neg) porcine oviduct epithelial cells was calculated via NanoString at 6, 12, 24, 36, and 48 hours (h) post infection. Data was analyzed by NanoString n solver software by comparing gene counts between neg and pos samples with grey cells indicating significant differences – $p < 0.05$.

Gene	6h pos	6h neg	Fold change	P value 6h	12h pos	12h neg	Fold change	P value 12h	24h pos	24h neg	Fold change	P value 24h	36h pos	36h neg	Fold change	P value 36h	48h pos	48h neg	Fold change	P value 48h
ADAMTS9	3.83	2.09	1.83	0.14	4.05	2.85	1.42	0.18	4.21	2.43	1.73	0.12	4.40	3.91	1.12	0.71	2.34	3.22	-1.37	0.42
CCL20	131.31	46.85	2.80	0.00	45.49	18.75	2.43	0.06	96.37	18.68	5.16	0.01	95.71	25.01	3.83	0.01	94.66	24.80	3.82	0.01
CCL4	25.07	4.56	5.49	0.00	12.90	4.20	3.07	0.02	11.03	3.79	2.91	0.04	11.03	5.92	1.86	0.01	5.95	2.35	2.53	0.05
CMPK2	2214.58	1848.02	1.20	0.27	2075.65	1409.90	1.47	0.09	2265.32	975.54	2.32	0.02	1824.59	705.20	2.59	0.00	1459.81	736.82	1.98	0.04
CSF3	16.57	10.38	1.60	0.11	13.86	7.17	1.93	0.01	14.65	8.94	1.64	0.14	18.13	13.26	1.37	0.28	18.50	14.67	1.26	0.06
CXCL10	989.90	624.11	1.59	0.21	656.52	170.26	3.86	0.05	1306.71	164.33	7.95	0.04	693.40	154.08	4.50	0.07	354.58	153.93	2.30	0.33
CXCL11	121.75	102.77	1.18	0.67	145.42	73.77	1.97	0.20	242.91	50.48	4.81	0.10	164.15	47.63	3.45	0.03	99.61	52.33	1.90	0.28
CXCL8	13008.71	10978.23	1.18	0.51	18026.86	18218.18	-1.01	0.97	14067.17	12594.78	1.12	0.68	11538.16	7695.53	1.50	0.16	7910.43	3974.95	1.99	0.01
CXCL9	20.08	23.18	-1.15	0.18	17.78	22.69	-1.28	0.15	25.90	23.33	1.11	0.52	22.39	26.96	-1.20	0.34	23.40	22.95	1.02	0.91
EBI3	5.97	6.44	-1.08	0.81	6.50	8.00	-1.23	0.40	9.44	6.92	1.36	0.09	8.97	8.03	1.12	0.58	7.04	6.15	1.14	0.65
Flt-3L	19.44	19.57	-1.01	0.96	16.95	14.88	1.14	0.22	17.11	16.52	1.04	0.86	20.13	17.66	1.14	0.54	19.03	14.89	1.28	0.17
HERC5	2086.67	1907.40	1.09	0.42	2350.44	1735.31	1.35	0.04	2424.81	1282.31	1.89	0.01	2129.95	1101.70	1.93	0.00	1765.67	1040.31	1.70	0.02
ICAM1	964.51	756.18	1.28	0.17	711.89	576.75	1.23	0.30	741.03	571.64	1.30	0.26	808.21	594.12	1.36	0.02	767.80	637.62	1.20	0.23
IL-1 alpha	575.69	421.99	1.36	0.01	534.35	442.03	1.21	0.21	551.51	446.34	1.24	0.22	550.82	466.80	1.18	0.20	573.02	398.16	1.44	0.01
IL-10	2.08	2.56	-1.23	0.63	1.99	1.92	1.03	0.91	1.17	1.65	-1.41	0.23	1.58	2.03	-1.29	0.55	1.22	1.69	-1.39	0.25
IL-15	77.25	72.68	1.06	0.75	126.43	83.09	1.52	0.11	219.30	136.80	1.60	0.01	187.75	135.45	1.39	0.09	193.21	174.77	1.11	0.63
IL-16	6.24	6.42	-1.03	0.94	7.12	5.85	1.22	0.38	8.61	8.76	-1.02	0.95	7.03	6.17	1.14	0.70	9.35	8.87	1.05	0.80
IL-1RA	22.02	21.60	1.02	0.89	23.40	16.90	1.39	0.07	28.56	19.23	1.49	0.02	31.60	30.69	1.03	0.89	46.92	36.43	1.29	0.31
IL-6	216.63	169.36	1.28	0.47	222.50	176.70	1.26	0.56	285.41	168.08	1.70	0.26	256.86	162.46	1.58	0.15	236.57	158.44	1.49	0.15
IL-7	36.49	31.23	1.17	0.32	32.48	26.86	1.21	0.31	50.96	27.49	1.85	0.02	49.34	26.74	1.84	0.08	40.87	29.74	1.37	0.15
MMP-2	368.09	347.19	1.06	0.75	278.45	309.55	-1.11	0.65	278.92	215.32	1.30	0.39	214.37	181.27	1.18	0.49	235.06	243.25	-1.03	0.89
MMP-9	26.88	21.85	1.23	0.67	17.38	15.88	1.09	0.83	10.86	12.01	-1.11	0.87	10.10	9.23	1.09	0.82	9.89	6.89	1.43	0.21
MX1	9379.78	8438.14	1.11	0.41	9657.67	6927.36	1.39	0.10	9238.90	4001.03	2.31	0.03	7679.56	3037.33	2.53	0.01	5610.78	2836.02	1.98	0.05
MX2	413.07	299.10	1.38	0.05	146.88	69.23	2.12	0.00	194.74	55.76	3.49	0.01	113.99	42.89	2.66	0.04	83.76	48.26	1.74	0.20
NEURL3	1.18	1.25	-1.06	0.78	1.06	1.26	-1.19	0.11	1.01	1.10	-1.08	0.25	1.05	1.43	-1.36	0.43	1.27	1.13	1.12	0.53
OAS2	2556.12	2308.47	1.11	0.73	2106.88	1615.02	1.30	0.44	1660.46	864.58	1.92	0.20	1706.04	712.33	2.40	0.05	1362.67	739.31	1.84	0.17
RANTES	95.47	59.58	1.60	0.09	99.99	34.66	2.88	0.05	224.87	41.48	5.42	0.02	192.52	25.94	7.42	0.00	118.92	29.17	4.08	0.00
SAA2	3.36	8.56	-2.55	0.02	7.37	6.84	1.08	0.77	4.84	5.81	-1.2	0.63	4.27	5.95	-1.39	0.20	4.64	4.57	1.02	0.96
TGF-b	1181.15	1123.74	1.05	0.86	791.91	856.27	-1.08	0.83	518.21	475.19	1.09	0.84	415.12	426.45	-1.03	0.93	447.65	512.29	-1.14	0.61
TNF-alpha	7.76	8.74	-1.13	0.53	7.82	9.04	-1.16	0.54	10.27	5.95	1.73	0.07	7.1	8.73	-1.23	0.69	5.62	7.95	-1.42	0.24
VEGF	2192.06	2037.22	1.08	0.51	1888.03	1788.25	1.06	0.69	2264.86	1874.13	1.21	0.21	2253.5	2244.13	1	0.98	2739.96	2848.92	-1.04	0.83
